# Bis(melaminium) tartrate dihydrate

**DOI:** 10.1107/S1600536809011143

**Published:** 2009-03-31

**Authors:** Hong Su, Yao-Kang Lv, Yun-Long Feng

**Affiliations:** a Zhejiang Key Laboratory for Reactive Chemistry on Solid Surfaces, Institute of Physical Chemistry, Zhejiang Normal University, Jinhua, Zhejiang 321004, People’s Republic of China

## Abstract

In the title compound, 2C_3_H_7_N_6_^+^·C_4_H_4_O_6_^2−^·2H_2_O, in which the complete anion is generated by crystallographic twofold symmetry, there are O—H⋯O, N—H⋯O and N—H⋯N hydrogen-bonding inter­actions between neighbouring moieties, forming layers parallel to the *bc* plane. In addition, π–π contacts [centroid–centroid distance = 3.6541 (9) Å] between the six-membered rings of the melamine cations are observed.

## Related literature

For general background, see: Row (1999 ▶); Krische & Lehn (2000 ▶); Sherrington & Taskinen (2001 ▶); Marchewka *et al.* (2003 ▶); Thushari *et al.* (2005 ▶). For related structures, see: Udaya Lakshmi *et al.* (2006 ▶). 
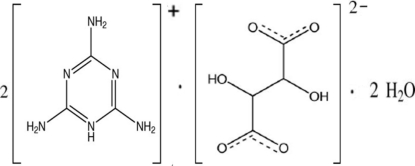


## Experimental

### 

#### Crystal data

2C_3_H_7_N_6_^+^·C_4_H_4_O_6_^2−^·2H_2_O
*M* *_r_* = 436.38
Monoclinic, 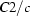

*a* = 7.6963 (9) Å
*b* = 21.955 (3) Å
*c* = 10.7405 (12) Å
β = 98.179 (6)°
*V* = 1796.4 (4) Å^3^
*Z* = 4
Mo *K*α radiation
μ = 0.14 mm^−1^
*T* = 296 K
0.26 × 0.22 × 0.12 mm

#### Data collection

Bruker APEXII area-detector diffractometer
Absorption correction: multiscan (*SADABS*; Sheldrick, 1996 ▶) *T*_min_ = 0.963, *T*_max_ = 0.980
13436 measured reflections
2047 independent reflections
1712 reflections with *I* > 2σ(*I*)
*R*_int_ = 0.027

#### Refinement

*R*[*F* ^2^ > 2σ(*F* ^2^)] = 0.037
*wR*(*F* ^2^) = 0.108
*S* = 1.00
2047 reflections
166 parameters
15 restraints
H atoms treated by a mixture of independent and constrained refinement
Δρ_max_ = 0.25 e Å^−3^
Δρ_min_ = −0.24 e Å^−3^

### 

Data collection: *APEX2* (Bruker, 2006 ▶); cell refinement: *SAINT* (Bruker, 2006 ▶); data reduction: *SAINT*; program(s) used to solve structure: *SHELXS97* (Sheldrick, 2008 ▶); program(s) used to refine structure: *SHELXL97* (Sheldrick, 2008 ▶); molecular graphics: *SHELXTL* (Sheldrick, 2008 ▶); software used to prepare material for publication: *SHELXTL*.

## Supplementary Material

Crystal structure: contains datablocks I, global. DOI: 10.1107/S1600536809011143/at2740sup1.cif

Structure factors: contains datablocks I. DOI: 10.1107/S1600536809011143/at2740Isup2.hkl

Additional supplementary materials:  crystallographic information; 3D view; checkCIF report

## Figures and Tables

**Table 1:** Hydrogen-bond geometry (Å, °)

*D*—H⋯*A*	*D*—H	H⋯*A*	*D*⋯*A*	*D*—H⋯*A*
O3—H3⋯O2^i^	0.870 (11)	1.802 (12)	2.6680 (14)	173.2 (16)
N1—H1*NA*⋯O1	0.884 (13)	2.271 (14)	3.0466 (19)	146.3 (16)
N1—H1*NB*⋯N4^ii^	0.879 (13)	2.151 (13)	3.0287 (19)	177.3 (17)
N2—H2*NA*⋯O3	0.889 (15)	2.093 (15)	2.8333 (16)	140.2 (14)
N2—H2*NA*⋯O1	0.889 (15)	2.190 (15)	2.9497 (18)	143.2 (14)
N3—H3*NA*⋯O1*W*^iii^	0.872 (14)	2.261 (19)	2.8609 (18)	125.9 (15)
N3—H3*NA*⋯O3	0.872 (14)	2.573 (16)	3.2241 (18)	132.2 (16)
N3—H3*NB*⋯N6^iv^	0.900 (14)	2.133 (14)	3.0313 (19)	176.2 (18)
N5—H5*NA*⋯O1*W*^v^	0.901 (13)	1.940 (14)	2.8148 (16)	163.2 (15)
N5—H5*NB*⋯O2^vi^	0.895 (13)	2.153 (15)	2.9581 (16)	149.4 (15)
O1*W*—H1*WA*⋯O1	0.858 (14)	1.852 (14)	2.6861 (16)	163.8 (17)
O1*W*—H1*WB*⋯O2^vii^	0.804 (13)	2.297 (15)	2.9738 (17)	142.3 (17)

Symmetry codes: (i) 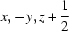; (ii) 
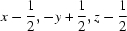
; (iii) 
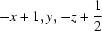
; (iv) 
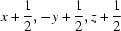
; (v) 
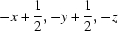
; (vi) 
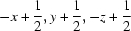
; (vii) 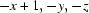.

**Fig. 1.**
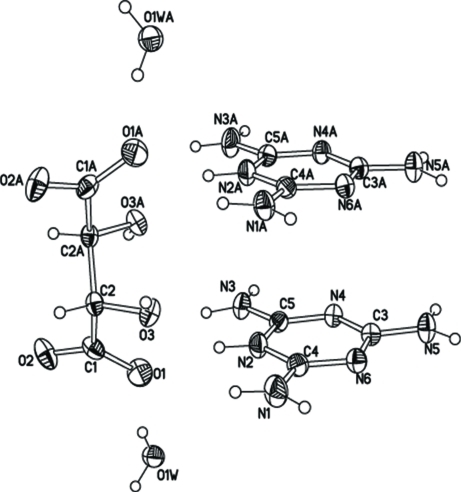
View of the molecule of (I), showing the atom-numbering scheme. Displacement ellipsoids plotted at 30% probability level. [The atoms labelled with 'A' are related to the center of inversion].

**Fig. 2.**
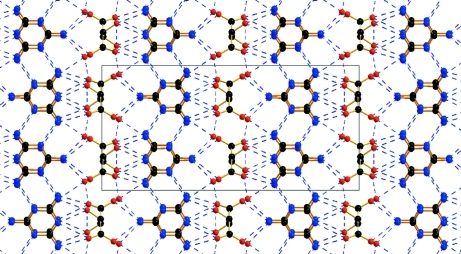
Packing diagram for compound (I). The O—H···O and O—H···N interactions are depicted by dashed lines.

## supplementary crystallographic information

### Comment

Melamine and its organic and inorganic counterparts can develop supramolecular assemblies *via* multiple hydrogen bonds (Row, 1999; Krische & Lehn, 2000; Sherrington & Taskinen, 2001; Marchewka *et al.*, 2003), while tartaric acid is a small organic molecule [C_4_H_4_O_6_] with a bewildering array of ligation possibilities (Thushari *et al.*, 2005). Herein we report the synthesis and crystal structure of the title compound (I).

In (I) (Fig. 1), the melaminium cations form infinite floors *via* N—H···N hydrogen bonds and the D-tartrate anions link pair with waters *via* O—H···O form floors lying between two floors of melaminium. Furthermore, the N—H···O hydrogen bonds connected the neighboring cations floors and anions floors is together into a three-dimensional network. We found that the architecture of compound (I) is similar to bis (melaminium) L– tartrate 2.5-hydrate (Udaya Lakshmi *et al.*, 2006) but not the same, which indicate that using different stereo-chemical configurations can give different three-dimensional arrangements. In addition, π–π contacts [centroid-centroid distance 3.6541 (9) Å] between the six-membered rings of the melamine moieties are observed.

### Experimental

Compound (I) is formed by hydrothermal reaction of D-tartaric acid (1.5 mmol) and Melamine (1 mmol) in 15 ml water for 2 days at 533 K.

### Refinement

The H atoms bonded to C atoms were positioned geometrically [C—H 0.96 Å *U*_iso_(H) = 1.2*U*_eq_(C)]. The H atoms bonded to O atoms were located in a difference Fourier maps and their positions were refined isotropically, with O—H distances fixed by O—H = 0.85 (2) Å and H ··· H = 1.30 (2) Å, their displacement parameters were set to 1.5*U*_eq_(O). The H atoms bonded to N atoms were located in a difference Fourier maps and their positions were refined isotropically, with N—H distances fixed by N—H = 0.90 (2) Å and H ··· H = 1.56 (2) Å, their displacement parameters were set to 1.2*U*_eq_(N).

### Crystal data

**Table:**

2C_3_H_7_N_6_^+^·C_4_H_4_O_6_^2^^−^·2H_2_O	*F*(000) = 920
*M**_r_* = 436.38	*D*_x_ = 1.621 Mg m^−^^3^
Monoclinic, *C*2/*c*	Mo *K*α radiation, λ = 0.71073 Å
Hall symbol: -C 2yc	Cell parameters from 4811 reflections
*a* = 7.6963 (9) Å	θ = 1.9–27.5°
*b* = 21.955 (3) Å	µ = 0.14 mm^−^^1^
*c* = 10.7405 (12) Å	*T* = 296 K
β = 98.179 (6)°	Block, colourless
*V* = 1796.4 (4) Å^3^	0.26 × 0.22 × 0.12 mm
*Z* = 4	

### Data collection

**Table:**

Bruker APEXII area-detector diffractometer	2047 independent reflections
Radiation source: fine-focus sealed tube	1712 reflections with *I* > 2σ(*I*)
graphite	*R*_int_ = 0.027
ω scans	θ_max_ = 27.5°, θ_min_ = 1.9°
Absorption correction: multi-scan (*SADABS*; Sheldrick, 1996)	*h* = −9→10
*T*_min_ = 0.963, *T*_max_ = 0.980	*k* = −27→28
13436 measured reflections	*l* = −13→13

### Refinement

**Table:**

Refinement on *F*^2^	Primary atom site location: structure-invariant direct methods
Least-squares matrix: full	Secondary atom site location: difference Fourier map
*R*[*F*^2^ > 2σ(*F*^2^)] = 0.037	Hydrogen site location: inferred from neighbouring sites
*wR*(*F*^2^) = 0.108	H atoms treated by a mixture of independent and constrained refinement
*S* = 1.00	*w* = 1/[σ^2^(*F*_o_^2^) + (0.059*P*)^2^ + 0.9299*P*] where *P* = (*F*_o_^2^ + 2*F*_c_^2^)/3
2047 reflections	(Δ/σ)_max_ < 0.001
166 parameters	Δρ_max_ = 0.25 e Å^−^^3^
15 restraints	Δρ_min_ = −0.24 e Å^−^^3^

### Special details

**Table:**

Geometry. All e.s.d.'s (except the e.s.d. in the dihedral angle between two l.s. planes) are estimated using the full covariance matrix. The cell e.s.d.'s are taken into account individually in the estimation of e.s.d.'s in distances, angles and torsion angles; correlations between e.s.d.'s in cell parameters are only used when they are defined by crystal symmetry. An approximate (isotropic) treatment of cell e.s.d.'s is used for estimating e.s.d.'s involving l.s. planes.
Refinement. Refinement of *F*^2^ against ALL reflections. The weighted *R*-factor *wR* and goodness of fit *S* are based on *F*^2^, conventional *R*-factors *R* are based on *F*, with *F* set to zero for negative *F*^2^. The threshold expression of *F*^2^ > σ(*F*^2^) is used only for calculating *R*-factors(gt) *etc*. and is not relevant to the choice of reflections for refinement. *R*-factors based on *F*^2^ are statistically about twice as large as those based on *F*, and *R*- factors based on ALL data will be even larger.

### Fractional atomic coordinates and isotropic or equivalent isotropic displacement parameters (Å^2^)

**Table:**

	*x*	*y*	*z*	*U*_iso_*/*U*_eq_	
O1	0.25143 (17)	0.05132 (5)	0.11300 (12)	0.0603 (3)	
O2	0.17077 (17)	−0.04576 (5)	0.08785 (10)	0.0535 (3)	
O3	0.15841 (13)	0.05730 (4)	0.33946 (9)	0.0394 (3)	
H3	0.158 (2)	0.0508 (7)	0.4193 (11)	0.047*	
N1	0.06055 (18)	0.17081 (6)	0.04393 (13)	0.0470 (3)	
H1NA	0.074 (2)	0.1308 (6)	0.0459 (17)	0.056*	
H1NB	−0.004 (2)	0.1902 (7)	−0.0177 (15)	0.056*	
N2	0.23582 (16)	0.17105 (6)	0.23551 (12)	0.0428 (3)	
H2NA	0.233 (2)	0.1306 (7)	0.2342 (16)	0.051*	
N3	0.4140 (2)	0.17049 (7)	0.42646 (15)	0.0558 (4)	
H3NA	0.407 (2)	0.1308 (7)	0.4247 (18)	0.067*	
H3NB	0.477 (2)	0.1917 (8)	0.4890 (16)	0.067*	
N4	0.32789 (15)	0.26210 (5)	0.33724 (11)	0.0373 (3)	
N5	0.22621 (18)	0.35022 (5)	0.24256 (11)	0.0451 (3)	
H5NA	0.157 (2)	0.3702 (8)	0.1812 (13)	0.054*	
H5NB	0.282 (2)	0.3704 (8)	0.3088 (13)	0.054*	
N6	0.13898 (15)	0.26268 (5)	0.13787 (10)	0.0353 (3)	
C1	0.18246 (18)	0.00368 (6)	0.14673 (13)	0.0392 (3)	
C2	0.09995 (16)	0.00520 (5)	0.26757 (11)	0.0308 (3)	
H2A	0.1329	−0.0309	0.3157	0.037*	
C3	0.23110 (16)	0.29059 (6)	0.23929 (11)	0.0338 (3)	
C4	0.14503 (16)	0.20248 (6)	0.13831 (13)	0.0352 (3)	
C5	0.32593 (17)	0.20212 (6)	0.33379 (13)	0.0386 (3)	
O1W	0.45183 (14)	0.06665 (5)	−0.06993 (11)	0.0465 (3)	
H1WA	0.391 (2)	0.0544 (8)	−0.0137 (16)	0.056*	
H1WB	0.535 (2)	0.0446 (8)	−0.0730 (17)	0.056*	

### Atomic displacement parameters (Å^2^)

**Table:**

	*U*^11^	*U*^22^	*U*^33^	*U*^12^	*U*^13^	*U*^23^
O1	0.0750 (8)	0.0567 (7)	0.0575 (7)	0.0020 (6)	0.0378 (6)	0.0128 (6)
O2	0.0879 (8)	0.0446 (6)	0.0312 (5)	0.0254 (5)	0.0197 (5)	0.0046 (4)
O3	0.0533 (6)	0.0362 (5)	0.0272 (5)	−0.0122 (4)	0.0010 (4)	0.0008 (4)
N1	0.0582 (8)	0.0324 (6)	0.0480 (8)	−0.0069 (5)	−0.0006 (6)	−0.0048 (5)
N2	0.0496 (7)	0.0278 (6)	0.0494 (7)	0.0002 (5)	0.0013 (5)	0.0020 (5)
N3	0.0641 (8)	0.0382 (7)	0.0591 (9)	0.0069 (6)	−0.0120 (7)	0.0110 (6)
N4	0.0433 (6)	0.0337 (6)	0.0333 (6)	0.0020 (4)	0.0003 (5)	0.0027 (4)
N5	0.0658 (8)	0.0288 (6)	0.0358 (7)	−0.0001 (5)	−0.0097 (6)	0.0001 (5)
N6	0.0434 (6)	0.0308 (6)	0.0310 (6)	−0.0022 (4)	0.0033 (5)	0.0001 (4)
C1	0.0454 (7)	0.0420 (8)	0.0320 (7)	0.0151 (6)	0.0118 (5)	0.0100 (5)
C2	0.0422 (7)	0.0264 (6)	0.0236 (6)	0.0017 (5)	0.0046 (5)	0.0031 (4)
C3	0.0395 (6)	0.0329 (7)	0.0291 (6)	−0.0004 (5)	0.0048 (5)	0.0012 (5)
C4	0.0366 (6)	0.0328 (7)	0.0372 (7)	−0.0028 (5)	0.0086 (5)	−0.0003 (5)
C5	0.0383 (6)	0.0359 (7)	0.0412 (7)	0.0022 (5)	0.0044 (5)	0.0051 (6)
O1W	0.0443 (6)	0.0467 (6)	0.0496 (6)	0.0051 (4)	0.0102 (5)	0.0143 (5)

### Geometric parameters (Å, °)

**Table:**

O1—C1	1.2497 (18)	N4—C5	1.3174 (18)
O2—C1	1.2530 (18)	N4—C3	1.3527 (16)
O3—C2	1.4170 (15)	N5—C3	1.3104 (18)
O3—H3	0.870 (11)	N5—H5NA	0.901 (13)
N1—C4	1.3215 (18)	N5—H5NB	0.895 (13)
N1—H1NA	0.884 (13)	N6—C4	1.3224 (18)
N1—H1NB	0.879 (13)	N6—C3	1.3583 (16)
N2—C4	1.3590 (18)	C1—C2	1.5242 (18)
N2—C5	1.3610 (18)	C2—C2^i^	1.531 (2)
N2—H2NA	0.889 (15)	C2—H2A	0.9600
N3—C5	1.3193 (18)	O1W—H1WA	0.858 (14)
N3—H3NA	0.872 (14)	O1W—H1WB	0.804 (13)
N3—H3NB	0.900 (14)		
			
C2—O3—H3	111.1 (11)	O2—C1—C2	116.11 (12)
C4—N1—H1NA	117.6 (12)	O3—C2—C1	110.08 (10)
C4—N1—H1NB	119.1 (12)	O3—C2—C2^i^	111.37 (8)
H1NA—N1—H1NB	123.3 (16)	C1—C2—C2^i^	108.42 (12)
C4—N2—C5	119.39 (13)	O3—C2—H2A	109.5
C4—N2—H2NA	119.1 (11)	C1—C2—H2A	109.3
C5—N2—H2NA	121.5 (11)	C2^i^—C2—H2A	108.2
C5—N3—H3NA	119.1 (13)	N5—C3—N4	117.12 (12)
C5—N3—H3NB	117.1 (12)	N5—C3—N6	117.29 (12)
H3NA—N3—H3NB	123.8 (17)	N4—C3—N6	125.59 (13)
C5—N4—C3	115.95 (12)	N1—C4—N6	120.66 (13)
C3—N5—H5NA	118.9 (11)	N1—C4—N2	117.72 (13)
C3—N5—H5NB	120.3 (11)	N6—C4—N2	121.62 (12)
H5NA—N5—H5NB	120.5 (15)	N4—C5—N3	120.17 (13)
C4—N6—C3	115.71 (11)	N4—C5—N2	121.69 (12)
O1—C1—O2	125.57 (13)	N3—C5—N2	118.14 (14)
O1—C1—C2	118.30 (13)	H1WA—O1W—H1WB	110.6 (16)
			
O1—C1—C2—O3	−15.91 (17)	C3—N6—C4—N1	−179.76 (12)
O2—C1—C2—O3	165.79 (11)	C3—N6—C4—N2	0.99 (18)
O1—C1—C2—C2^i^	106.14 (12)	C5—N2—C4—N1	179.33 (13)
O2—C1—C2—C2^i^	−72.17 (12)	C5—N2—C4—N6	−1.4 (2)
C5—N4—C3—N5	177.80 (13)	C3—N4—C5—N3	−178.79 (13)
C5—N4—C3—N6	−2.34 (19)	C3—N4—C5—N2	1.85 (19)
C4—N6—C3—N5	−179.21 (12)	C4—N2—C5—N4	−0.1 (2)
C4—N6—C3—N4	0.93 (18)	C4—N2—C5—N3	−179.50 (13)

Symmetry codes: (i) −*x*, *y*, −*z*+1/2.

### Hydrogen-bond geometry (Å, °)

**Table:**

*D*—H···*A*	*D*—H	H···*A*	*D*···*A*	*D*—H···*A*
O3—H3···O2^ii^	0.87 (1)	1.80 (1)	2.6680 (14)	173 (2)
N1—H1NA···O1	0.88 (1)	2.27 (1)	3.0466 (19)	146 (2)
N1—H1NB···N4^iii^	0.88 (1)	2.15 (1)	3.0287 (19)	177 (2)
N2—H2NA···O3	0.89 (2)	2.09 (2)	2.8333 (16)	140 (1)
N2—H2NA···O1	0.89 (2)	2.19 (2)	2.9497 (18)	143 (1)
N3—H3NA···O1W^iv^	0.87 (1)	2.26 (2)	2.8609 (18)	126 (2)
N3—H3NA···O3	0.87 (1)	2.57 (2)	3.2241 (18)	132 (2)
N3—H3NB···N6^v^	0.90 (1)	2.13 (1)	3.0313 (19)	176 (2)
N5—H5NA···O1W^vi^	0.90 (1)	1.94 (1)	2.8148 (16)	163 (2)
N5—H5NB···O2^vii^	0.90 (1)	2.15 (2)	2.9581 (16)	149 (2)
O1W—H1WA···O1	0.86 (1)	1.85 (1)	2.6861 (16)	164 (2)
O1W—H1WB···O2^viii^	0.80 (1)	2.30 (2)	2.9738 (17)	142 (2)

Symmetry codes: (ii) *x*, −*y*, *z*+1/2; (iii) *x*−1/2, −*y*+1/2, *z*−1/2; (iv) −*x*+1, *y*, −*z*+1/2; (v) *x*+1/2, −*y*+1/2, *z*+1/2; (vi) −*x*+1/2, −*y*+1/2, −*z*; (vii) −*x*+1/2, *y*+1/2, −*z*+1/2; (viii) −*x*+1, −*y*, −*z*.
